# Impacts of Biogas Slurry Fertilization on Arbuscular Mycorrhizal Fungal Communities in the Rhizospheric Soil of Poplar Plantations

**DOI:** 10.3390/jof8121253

**Published:** 2022-11-27

**Authors:** Xing-Ye Yu, Bao-Teng Wang, Long Jin, Hong-Hua Ruan, Hyung-Gwan Lee, Feng-Jie Jin

**Affiliations:** 1 Co-Innovation Center for Sustainable Forestry in Southern China, College of Biology and the Environment, Nanjing Forestry University, 159 Longpan Road, Nanjing 210037, China; 2 Cell Factory Research Centre, Korea Research Institute of Bioscience & Biotechnology (KRIBB), Daejeon 34141, Republic of Korea

**Keywords:** arbuscular mycorrhizal fungi, poplar plantations, plant–microbe interactions, rhizospheric soil, diversity

## Abstract

The majority of terrestrial plants are symbiotic with arbuscular mycorrhizal fungi (AMF). Plants supply carbohydrates to microbes, whereas AMF provide plants with water and other necessary nutrients—most typically, phosphorus. Understanding the response of the AMF community structure to biogas slurry (BS) fertilization is of great significance for sustainable forest management. This study aimed to look into the effects of BS fertilization at different concentrations on AMF community structures in rhizospheric soil in poplar plantations. We found that different fertilization concentrations dramatically affected the diversity of AMF in the rhizospheric soil of the poplar plantations, and the treatment with a high BS concentration showed the highest Shannon diversity of AMF and OTU richness (Chao1). Further analyses revealed that Glomerales, as the predominant order, accounted for 36.2–42.7% of the AMF communities, and the relative abundance of Glomerales exhibited negligible changes with different BS fertilization concentrations, whereas the order Paraglomerales increased significantly in both the low- and high-concentration treatments in comparison with the control. Furthermore, the addition of BS drastically enhanced the relative abundance of the dominant genera, *Glomus* and *Paraglomus*. The application of BS could also distinguish the AMF community composition in the rhizospheric soil well. An RDA analysis indicated that the dominant genus *Glomus* was significantly positively correlated with nitrate reductase activity, while *Paraglomus* showed a significant positive correlation with available P. Overall, the findings suggest that adding BS fertilizer to poplar plantations can elevate the diversity of AMF communities in rhizospheric soil and the relative abundance of some critical genera that affect plant nutrient uptake.

## 1. Introduction

Terrestrial plants are commonly found to have symbiotic relationships with mycorrhizal fungi, among which arbuscular mycorrhizal fungi (AMF) are the most abundant. AMF belong to the phylum Glomeromycota and are obligate plant symbionts [1]. AMF provide plants with phosphorus and other essential nutrients in exchange for carbohydrates, while they can also protect plants from drought and pathogens [2,3,4]. AMF are not host-specific and can combine with plant roots to form enormous underground networks. Because hyphal exudates of AM fungi dissolve more mineral phosphorus in the soil than root exudates alone, this suggests that AMF significantly enhance plant phosphorus uptake by improving soluble P release [5,6]. In addition to their effect on phosphorus, AMF can also improve plant establishment and boost the intake of water and other nutrient, including N and several essential mineral elements [7,8]. Moreover, AMF are also helpful in protecting plants from abiotic and biotic stresses, and they are beneficial for soil structure due to their ability to lead to aggregate formation [9,10]. Since AMF perform root-like functions, they may act as a functional extension of the root system.

Some studies have shown that the presence of AMF mycelia may change the composition of bacterial communities [11,12]. AMF mycelial exudates were found to play a crucial role in soil bacterial growth and changes in community structure [13,14]. This evidence showed that the mycelial exudates extracted from AMF can promote bacterial growth and alter the composition of bacterial communities. On AMF hyphae, several bacterial groups with higher abundance can be found, which would indirectly affect plant nutrient absorption and growth.

In forest management practice, fertilization is an efficient approach to preserving soil fertility. Different fertilization methods often have considerable influences on soil microbial populations and soil enzyme activity, and they can boost soil fertility and contribute to the long-term sustainability of forest ecosystems. However, frequent fertilization, especially with inorganic N and P fertilizers, can raise soil N and P content, potentially causing negative ecological consequences for terrestrial ecosystems, such as soil acidification, eutrophication, and biodiversity loss [15,16].

Several recent studies showed that the addition of biogas slurry (BS) as an organic fertilizer efficiently maintained the sustainability of agricultural soil while enhancing crop yields [17,18,19]. As an organic soil amendment, BS is composed of liquid mixtures of the methane produced by animal manure through anaerobic fermentation [20,21]. This nutrient-rich slurry has the potential to improve the physical, chemical, and biological properties of soil, such as by adjusting the soil pH, enhancing the soil’s aggregation, and boosting the soil’s total N and organic matter content, among other changes [22,23]. These changes in soil physical and chemical parameters may potentially impact the soil microbial biomass, microbial community composition, and soil enzyme activities [24,25,26].

It has been reported that on some agricultural lands, an increase in available nutrients due to fertilization also affects AMF communities, as it may lead to a decrease in these symbionts; that is, when soil nutrients are not limited, plants allocate resources to other restricted components, resulting in a reduction in fine roots and mycorrhizal structures [27]. Furthermore, fungi themselves are also nutrient-limited and sensitive to changes in soil properties [28,29]. Increased soil N concentrations caused by N fertilizer have been demonstrated in studies to diminish AMF biomass and alter AMF species richness and diversity in some terrestrial environments [30,31]. In addition, the effect of N fertilizer on AMF communities was also potentially related to soil P availability [32]. The rhizosphere is a highly active and dynamic interface between microorganisms and plants for nutrient exchange through the soil, where microbes and plant roots interact. The composition of microbial communities in rhizospheric soil is determined by the interactions between agricultural/forest management practices and host selection [33]; hence, studying changes in the structure of microbial communities, including AMF, in rhizospheric soil is essential. Field experiments on the interaction between P fertilizer level and AMF colonization in tomato roots showed that P fertilizer supply increased P uptake and plant biomass, but had no influence on AMF root colonization, diversity, or community structure [34]. According to another study, a moderate P input caused changes in the AMF community composition, although AMF richness decreased only after both P and N were added [35]. As BS may provide sufficient nutrients, such as organic N and P, simultaneously to the soil, it can help make up for the shortage of these nutrients in the soil. However, the effects of BS fertilization on AMF community structure and diversity have not been well studied. These results suggest that rhizospheric AMF may respond differently to nutrient additions. However, our understanding of how fertilizers affect this important symbiotic fungus is still limited, especially in terms of the effects of organic fertilizers, such as BS, on AMF. Therefore, understanding the changes in the structure and composition of rhizospheric AMF after fertilization may have important implications for the use of AMF to improve soil nutrient availability.

To study the potential effects of nutrient deposition on soil microbial communities and diversity, field trials with different fertilization strategies were started in 2012 in Dongtai poplar plantations located in a coastal area of eastern China [36,37]. In this study, the response of the composition and diversity of AMF communities to BS fertilization in rhizospheric soil was explored, and correlations among the AMF species diversity, dominant taxa, and soil characteristics were determined. It was hypothesized that BS fertilization would greatly alter the AMF community structure and increase the AMF diversity and species richness in the rhizospheric soil of poplar plantations. We anticipated that our findings would contribute to a clearer understanding of the mechanisms by which BS fertilizer can boost the growth and yields of poplar plantations.

## 2. Materials and Methods

### 2.1. Description of the Experimental Site and Soil Sampling

The experimental site was located in a poplar plantation on a forest farm in Jiangsu Province, Eastern China (32°52′ N, 120°49′ E). This forest farm has a subtropical climate, with an annual average temperature of 13.7 °C, precipitation of 1051 mm, and soil pH that is slightly alkaline [38,39].

In 2012, experiments were started in a 10-year-old poplar plantation (*Populus deltoids* cv. ‘I-35’) with consistent site characteristics and management histories [39]. Three replicate blocks were employed in a randomized block design. Each block consisted of three distinct treatment plots (20 m × 20 m). The poplar trees planted in the plots were spaced at a 5 m by 5 m interval with a separation of at least 500 m between any two neighboring blocks. Every year in May, August, and October since 2012, quantified biogas was sprayed uniformly into the ground of each block in the three different treatment plots. In September 2018, samples were collected from the upper layer (0–20 cm) of the rhizospheric soil of the poplar plantations in three different treatment plots: (1) Con—no treatment, (2) Low—biogas slurry applied at the rate of 250 m^3^ ha^−1^ yr^−1^, and (3) High—biogas slurry treatment at the rate of 375 m^3^ ha^−1^ yr^−1^.

### 2.2. Determination of Soil Physical and Chemical Properties

Rhizospheric soil samples were randomly selected from the nine treatment plots, mixed separately, sieved through 2 mm pores, and then used to analyze the DNA and the physical and chemical properties. The specific methodologies for assessing the physicochemical parameters of the rhizospheric soil were described in previous studies [37,39], and results are shown in Table S1. 

### 2.3. Genomic DNA Extraction

The FastDNA Spin Kit for Soil (MPbio, Carlsbad, CA, USA) was employed to extract the genomic DNA from the poplar rhizospheric soil samples. The concentration of isolated genomic DNA was quantified using a NanoDrop 2000C (Thermo Fisher Scientific, Waltham, MA, USA), and the genomic DNA’s integrity was evaluated by using a 0.8% (*w*/*v*) agarose gel electrophoresis.

### 2.4. PCR Amplification, Sequencing, and Data Processing

Amplification of the 18S rRNA gene was carried out by using specific PCR primers (AMF-F: 5′-AAGCTCGTAGTTGAATTTCG-3′ and AMF-R: 5′-CCCAACTATCCCTATTAATCAT-3′) for the analysis and identification of AMF communities [40]. The primer set, which included a unique 12-bp barcode for each sample, was chosen due to its repeatability and capacity to correctly reflect the AMF community composition [41], and it also included a unique 12-bp barcode for each sample. We used a BioRad S1000 (Bio-Rad Laboratories, Hercules, CA, USA) for PCR amplification, 1% agarose gel electrophoresis to detect the PCR products, and GeneTools Analysis Software (Version 4.03.05.0, SynGene) to mix in equidensity ratios. Using an EZNA Gel Extraction Kit (Omega Bio-Tek, Doraville, GA, USA), the mixed PCR products were further purified. To make sequencing libraries, we employed a NEBNext^®^ Ultra™ DNA Library Prep Kit for Illumina^®^ (New England Biolabs, Ipswich, MA, USA). An Agilent Bioanalyzer 2100 system (Agilent Technologies, Waldbron, Germany) and a Qubit@ 2.0 Fluorometer (Thermo Fisher Scientific, Waltham, MA, USA) were used to assess the library’s quality before it was sequenced on the Illumina Hiseq2500 platform [42].

Quality filtering on the paired-end raw reads was conducted under specific filtering circumstances to get clean high-quality reads according to the Trimmomatic Quality Control method [43]. Based on their unique barcodes and primers, the Mothur software (V1.35.1) was used to assign sequences to each sample, and then the primers and barcodes were deleted. The Usearch software was used to perform DNA sequence analysis. Sequences with ≥97% similarity were allocated to the same operational taxonomic unit (OTU) [44]. Singleton OTUs and chimera sequences were eliminated during the clustering process. OTU abundance data were normalized using a standard sequence number, and alpha and beta diversity analyses were performed by using the normalized data output. The raw sequencing data were deposited in the Sequence Read Archive (SRA) of the National Center for Biotechnology Information (NCBI) under the Bioproject PRJNA672251, with the AMF 18S rRNA accession numbers SRR19577676–SRR19577684.

### 2.5. Statistical Analysis

The Shannon diversity and Chao1 indices were employed to determine the alpha diversity of the AMF communities in the rhizospheric soil, while the phylogenetic diversity index was used to determine the phylogenetic diversity. All of these indices were computed with the QIIME software (ver.1.9.1) and displayed with the R software. The Bray–Curtis index was used to calculate the distance between the samples. Non-parametric multivariate analysis of variance (Adonis) was used to determine whether the differences between groups were significant. The relationships between the AMF community composition and environmental factors were examined by using redundancy analysis (RDA). In addition, we investigated the relative abundances of AMF orders and genera among the treatments with ANOVA and Tukey’s honestly significant difference (HSD) test. We used the R software to run all of the aforementioned analyses and provided the results as mean values and standard errors for each treatment. *p* < 0.05 was used to indicate a statistically significant difference between treatments. 

## 3. Results

### 3.1. Effect of Biogas Slurry Fertilization on the α-Diversity of AMF Communities in Rhizospheric Soil

**Figure 1:**
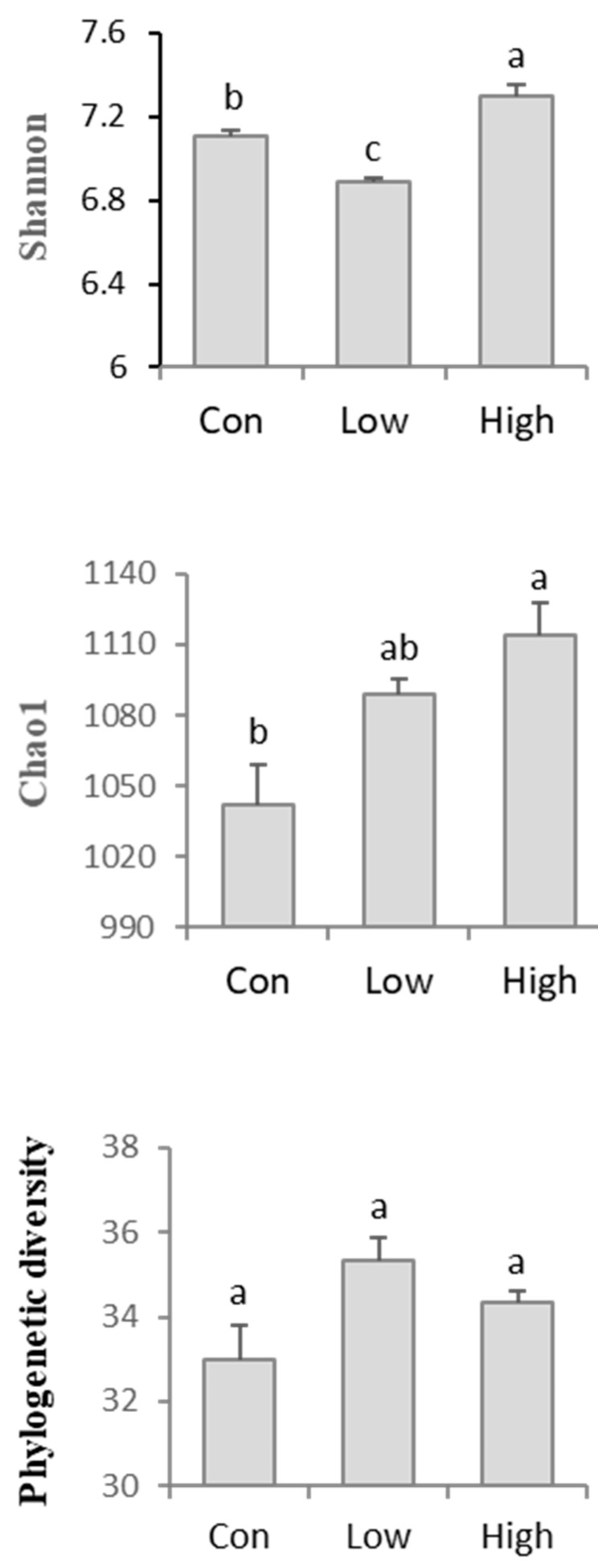
Alpha diversity analysis of the AMF communities in rhizospheric soil. The Shannon diversity, richness (Chao1), OTU number, and phylogenetic diversity among the different treatments were investigated and compared. Con—no treatment; Low—biogas slurry (250 m^3^ ha^−1^ yr^−1^) treatment; High—biogas slurry (375 m^3^ ha^−1^ yr^−1^) treatment. Error bars represent the standard errors of three replications. Different lowercase letters represent significant (*p* < 0.05) differences between treatments (*n* = 3).

The number of AMF OTUs in the rhizospheric soil was calculated under different BS concentrations. According to a Venn diagram analysis, the unique AMF OTU numbers were in the following sequence: High > Low > Con (Figure S1). When compared with the control, the high-concentration BS treatment considerably (*p* < 0.05) increased the Shannon diversity and Chao1 index of the AMF in the rhizospheric soil (Figure 1; Table S2). However, the low-concentration BS fertilization significantly (*p* < 0.05) decreased the Shannon diversity index. In addition, the BS fertilizer had no substantial influence on the phylogenetic diversity. An analysis of the molecular variance (AMOVA) was used to evaluate the degree of genetic differentiation between groups (Table S3).

### 3.2. Effect of Biogas Slurry Fertilization on the AMF Community Composition

**Figure 2:**
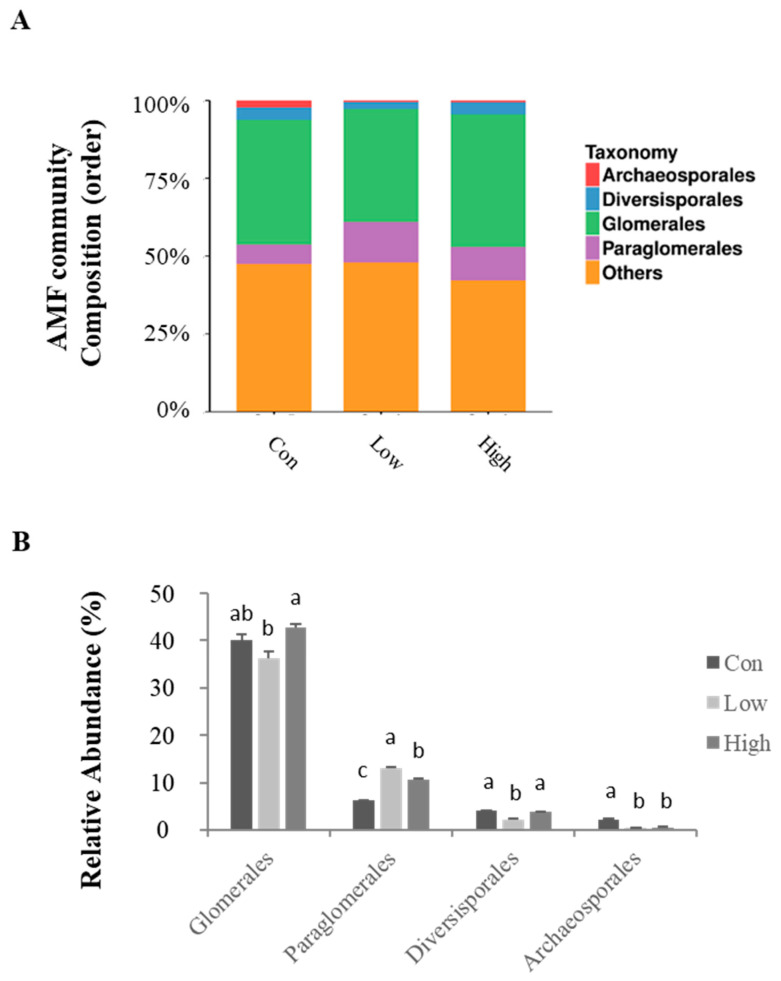
Relative abundance of the AMF community composition in rhizospheric soil at the order level. (**A**) The distributions of AMF community compositions at the order level. (**B**) Relative abundance of the AMF community composition at the order level. Con—no treatment; Low—biogas slurry (250 m^3^ ha^−1^ yr^−1^) treatment; High—biogas slurry (375 m^3^ ha^−1^ yr^−1^) treatment. Error bars represent the standard errors of three replications. Significant (*p* < 0.05) differences between the treatments are shown by different lowercase letters.

**Figure 3:**
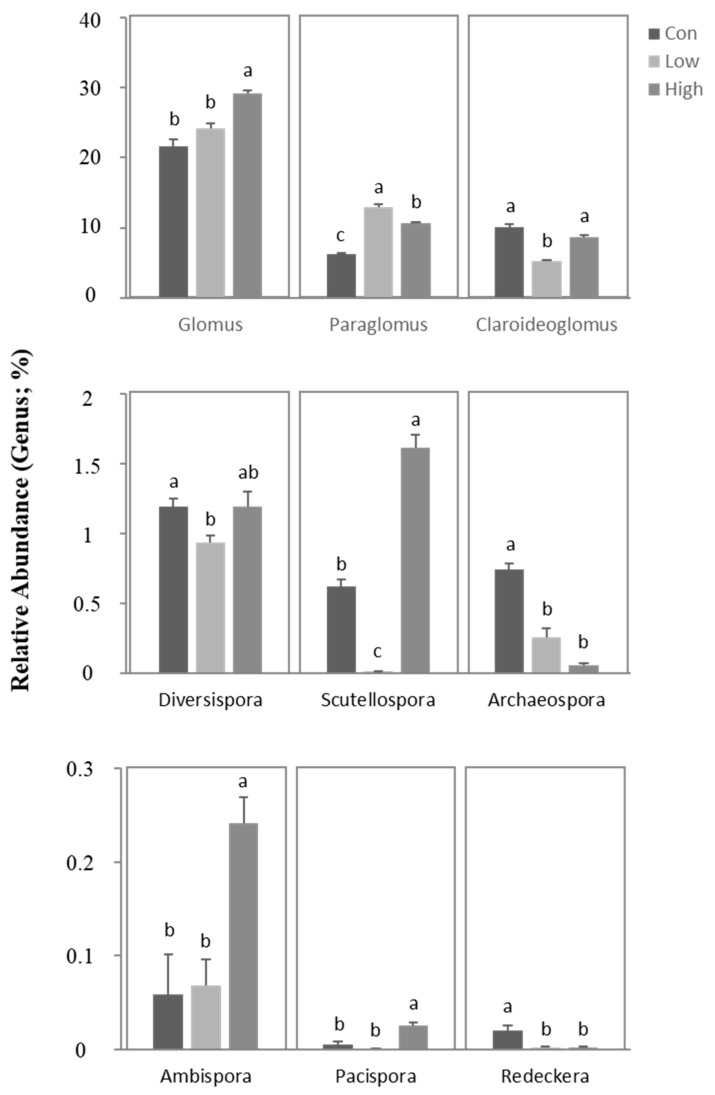
Relative abundance of the AMF community composition in rhizospheric soil at the genus level. Con—no treatment (control); Low—biogas slurry (250 m^3^ ha^−1^ yr^−1^) treatment; High—biogas slurry (375 m^3^ ha^−1^ yr^−1^) treatment. Error bars represent the standard errors of three replications. Different lowercase letters indicate significant (*p* < 0.05) differences between the treatments.

The composition and abundance of the AMF communities in the rhizospheric soil were investigated at the order (Figure 2 and Table S4) and genus (Figure 3 and Table S5) levels. Glomerales, as the predominant order, accounted for 36.2–42.7% of the AMF communities, and its relative abundance did not change significantly when BS fertilizer was added. By contrast, another major dominant order, Paraglomerales, increased significantly (*p* < 0.05) with both the low- and high-concentration treatments in comparison with the control (Figure 2B). In addition, the application of BS significantly (*p* < 0.05) reduced the relative abundance of Archaeosporales in comparison with the control (Figure 2B).

**Figure 4:**
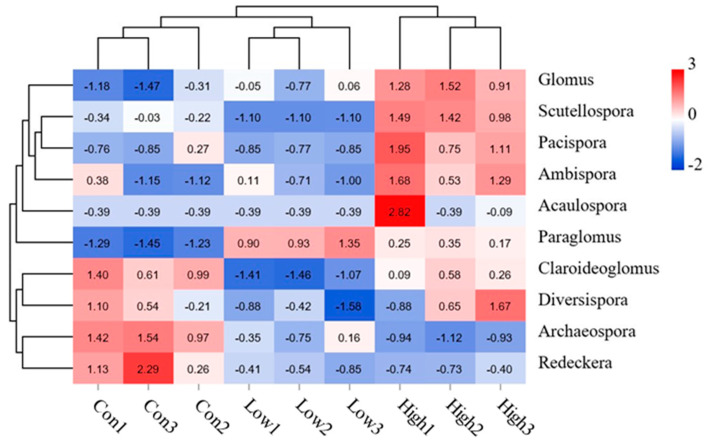
Heatmap analysis of the relative abundance of AMF genera in rhizospheric soil treated with different concentrations of biogas slurry.

At the genus level, *Glomus*, *Paraglomus*, and *Claroideoglomus* were found to be the dominant genera in the rhizospheric soil of the poplar plantation, accounting for 21.6–29.1%, 6.2–13.0%, and 5.2–10.1% of the total sequences, respectively. The addition of BS significantly (*p* < 0.05) improved the relative abundance of the dominant genera, *Glomus* and *Paraglomus* (Figure 3 and Figure 4). Furthermore, the high-concentration BS treatment significantly (*p* < 0.05) enhanced the relative abundance of *Scutellospora*, *Ambispora*, and *Pacispora*, among which the relative abundance of *Scutellospora* was drastically decreased by the low-concentration BS treatment. In contrast, the relative abundance of *Archaeospora* and *Redeckera* was significantly (*p* < 0.05) reduced with BS fertilization. In addition to these, fertilization with low-concentration BS also significantly (*p* < 0.05) reduced the abundance of *Claroideoglomus* and *Diversispora*, whereas their abundance was not significantly altered by BS fertilization at the high concentration in comparison with the control (Figure 3 and Figure 4). Principal coordinates analyses (PCoAs) based on the Bray–Curtis distance revealed that the duplicate samples were clustered together and clearly differentiated from the other treatments (Figure S2).

### 3.3. Relationships between AMF Community Composition and Soil Parameters

**Figure 5:**
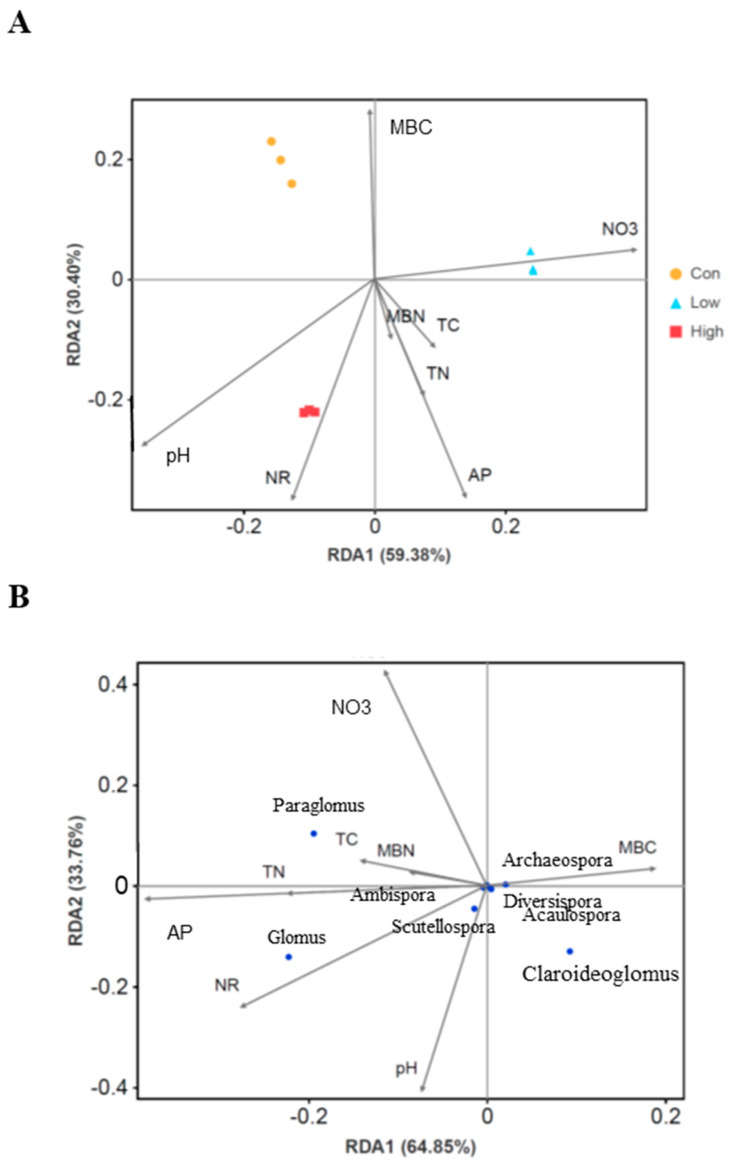
Redundancy analysis (RDA) between the AMF community composition in rhizospheric soil and environmental factors in poplar plantations treated with different concentrations of BS. (**A**) The RDA showed the relationship between the AMF community compositions based on the OTU abundance and environmental factors. (**B**) RDA for the correlations between AMF genera and environmental factors. Eight environmental factors were investigated for their correlations with AMF community composition. Abbreviations: MBC, microbial biomass carbon in rhizospheric soil; MBN, microbial biomass nitrogen in rhizospheric soil; TC, total carbon; TN, total nitrogen; NO_3_—, nitrate–nitrogen; AP, available phosphorus; NR, nitrate reductase.

Redundancy analysis (RDA) was used to assess the relationships between environmental factors and AMF communities in the poplar plantation’s rhizospheric soil when treated with various BS concentrations (Figure 5A). With the BS treatments, the duplicated samples of each treatment for the AMF communities could be easily separated from one another. The RDA axes 1 and 2 could explain 59.38% and 30.40%, respectively, of the variation in AMF communities across samples. The length of the line is proportional to the strength of the correlation with the ordination, and the vector represents the direction of the largest variation. The positive and negative correlations between AMF community composition and different environmental parameters were studied. The results showed that pH, NR activity, and AP were positively correlated with AMF composition in rhizosphere soil treated with high-concentration BS. NO_3_^−^-N and MBC were positively correlated with AMF composition in low-concentration BS and control treatments, respectively, whereas they were negatively correlated with AMF composition in the high-concentration BS treatment (Figure 5A).

**Figure 6:**
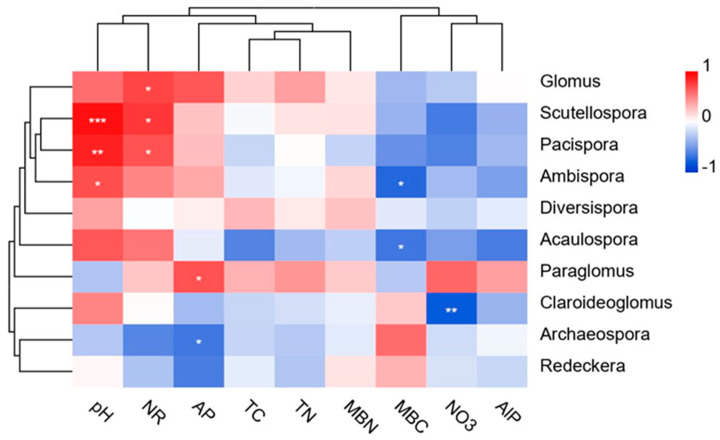
Heatmap analysis of the relationships between the AMF genera and environmental factors. Significant correlations are shown by * *p* < 0.05, ** *p* < 0.01, and *** *p* < 0.001.

RDA was used to further test the correlations between some major AMF genera and environmental factors (Figure 5B). The two axes of the ordination in the RDA explained the genus–environment relationship by 64.85% and 33.76%, respectively. The dominant genus, *Glomus,* displayed a positive correlation with NR, AP, and pH, while *Paraglomus* was positively correlated with NO_3_^−^N, AP, TN, and NR. The third most dominant genus, *Claroideoglomus,* showed a positive correlation with MBC and pH (Figure 5B). A further heatmap analysis based on the relationship of the AMF genera with the environmental factors revealed that: *Scutellospora*, *Ambispora*, and *Pacispora* had a significantly (*p* < 0.05) positive correlation with pH; *Glomus*, *Scutellospora*, and *Pacispora* were also significantly (*p* < 0.05) positively correlated with NR activity; *Ambispora*, *Acaulospora*, and *Claroideoglomus* displayed a significantly negative correlation with MBC and NO_3_^−^N, respectively. In addition, *Paraglomus* exhibited a significantly positive correlation with AP, whereas *Archaeospora* was strongly negatively correlated with AP (Figure 6).

## 4. Discussion

Fertilization is a typical method of forest management for improving forest productivity. In this study, the application of a high-concentration BS fertilizer significantly increased the Shannon diversity and species richness of AMF, while the low-concentration BS treatment decreased the Shannon diversity of AMF in the rhizospheric soil of poplar plantations. Furthermore, the application of BS significantly improved the relative abundance of the dominant genera, *Glomus* and *Paraglomus*, both of which play a crucial role in plant nutrient absorption. These findings suggest that BS fertilization can increase the diversity of AMF communities and change their structure in rhizospheric soil in poplar plantations.

In previous studies of physical and chemical properties, the application of BS fertilizer significantly improved the available P and led to a downward trend in the C/N ratio in the rhizospheric soil (Table S1), since BS is known to supply sufficient N and P [45]. Correspondingly, in our study, the low-concentration BS treatment significantly reduced the Shannon diversity of AMF (Figure 1). However, in comparison with the control, the high-concentration BS treatment dramatically elevated the Shannon diversity and species richness of AMF in the rhizospheric soil of poplar plantations. These results suggest that the application of organic fertilizers, such as BS, can significantly improve the diversity of AMF communities in rhizosphere soil under the conditions of appropriate fertilization concentrations. This is consistent with our findings in an earlier study, which showed that BS fertilization had a greater impact on the diversity of fungal communities in rhizospheric soil than bacteria, and a high-concentration BS treatment significantly raised the Shannon diversity of the fungal community in the rhizospheric soil [37]. Furthermore, although BS treatment did not significantly change the content of the total nitrogen in rhizospheric soil, the application of low-concentration BS significantly increased the nitrate nitrogen (Table S1).

According to our study, the AMF OTUs in poplar rhizospheric soil were mostly classified into four orders, nine families, and nine genera. At the level of the order, these OTUs belonged mainly to Glomerales (36.2–42.7%), Paraglomerales (6.2–13.0%), Diversisporales (2.2–4.0%), and Archaeosporales (0.5–2.2%). At the genus level, *Glomus* (21.6–29.1%), *Paraglomus* (6.2–13.0%), *Claroideoglomus* (5.2–10.1%), and *Diversispora* (0.9–1.2%) accounted for a considerable proportion, of which *Glomus*, *Paraglomus*, and *Claroideoglomus* were dominant. The relative abundance of the genera *Glomus* and *Paraglomus* was dramatically elevated with the addition of BS fertilizer (Figure 3), suggesting that organic BS fertilizer can significantly increase the abundance of dominant AMF genera in poplar rhizospheric soil. These major dominant AMF genera—especially *Glomus*—were also frequently detected in the rhizosphere of many other plant species [46,47,48], and some studies have shown that organic fertilizers can improve abundance of *Glomus* species in agricultural soils [49,50].

The addition of organic fertilizer activated more AMF species in rhizospheric soil. In comparison with the application of traditional inorganic fertilizer, organic fertilizer increased soil fertility and activated a wider range of soil microorganisms [51,52]. That is because conventional inorganic fertilizers, such as inorganic N and P fertilizers, can be directly absorbed by plants, while organic fertilizers, such as BS, provide more organic matter that needs to be decomposed and transformed by a variety of microorganisms before plants can use it. Therefore, organic fertilizers are more conducive to microbial growth and have a positive impact on the diversity of soil microorganisms, especially fungal communities, including AMF. Interestingly, according to our results, the available P and nitrate nitrogen in soil significantly increased with the addition of BS fertilizer. Furthermore, we also found that the dominant genus, *Glomus,* was significantly positively correlated with NR activity, while *Paraglomus* showed a significantly positive correlation with available P (Figure 5B and Figure 6). On the contrary, inorganic N and P fertilizers could not stimulate and induce greater soil microbial diversity, but they resulted in a reduction in microbial community diversity due to the single nutrients provided. It was reported that some species of *Glomus* were relatively more abundant with treatments using increased organic fertilizer, while the abundance of *Paraglomus* was higher with a treatment using a low-concentration organic fertilizer [53], which was also consistent with our current research findings (Figure 3). The responses of different AMF species to organic fertilizer were significantly different, indicating that the life strategies of AMF differed to some extent. Furthermore, studies have shown that *Glomus* is more resistant and adaptable to ecological disturbances and, therefore, often plays a critical role in performing ecological functions, such as mediating the interactions of AMF species [54,55]. This study indicated that genetic exchange might occur via fusion of the hyphae from distinct *Glomus* species, thus paving the path for further research on the vegetative compatibility between AMF populations [54]. Moreover, in a recent work, we found that applying inorganic nitrogen fertilizer to mature poplar plantations markedly decreased the Shannon index of fungi in rhizospheric soil [36]. In contrast, the addition of organic fertilizers, such as biogas slurry, effectively boosted the Shannon diversity of fungal communities [37], which was consistent with the results for AMF diversity in this study. Our data support the hypothesis that the application of biogas slurry might alter the composition of the AMF communities in rhizospheric soil and raise the relative abundance of major dominant AMF genera, hence further illuminating the mechanism the benefit to plant growth provided by the application of biogas slurry.

## 5. Conclusions

We investigated the impacts of organic BS fertilizer on AMF communities. The application of BS significantly increased the Shannon diversity and species richness of AMF communities in the rhizospheric soil of poplar plantations. BS fertilization also resulted in the activation of more OTUs in the poplar rhizospheric soil and significantly enhanced the abundance of the dominant genera, *Glomus* and *Paraglomus*, suggesting that BS treatment might lead to more complicated interspecific interactions in the rhizospheric soil of poplar plantations. Furthermore, the changes in the physical and chemical properties of the poplar rhizospheric soil caused by BS fertilization were also significantly correlated with the composition of the AMF community: The dominant genus, *Glomus,* was significantly positively correlated with NR activity, while *Paraglomus* showed a significantly positive correlation with AP.

## Data Availability

Not applicable.

## Supplementary Materials

The following supporting information can be downloaded at: https://www.mdpi.com/article/10.3390/jof8121253/s1, Figure S1: Venn diagram depicting the relationships between treatments; Figure S2: Principal coordinates analysis on AMF community structure in rhizosphere soil of poplar plantations treated with different concentrations of biogas slurry; Table S1: Effects of biogas slurry treatments on the physical, chemical, and biological characteristics of rhizospheric soil; Table S2: Effect of biogas slurry fertilization on α-diversity of AMF communities; Table S3: Genetic differentiation of AMF communities between groups by analysis of molecular variance (AMOVA); Table S4: Relative abundance of AMF community composition at the order level; Table S5: Relative abundance of AMF community composition at the genus level.
