# Supplementary material for: Impacts of Biogas Slurry Fertilization on Arbuscular Mycorrhizal Fungal Communities in the Rhizospheric Soil of Poplar Plantations
Source: J Fungi (Basel). 2022 Nov 27;8(12):1253. doi: 10.3390/jof8121253 (PMC9782214; doi:10.3390/jof8121253)
Supplement: Supplementary file 1 [file jof-08-01253-s001.zip › jof-1968655-supplementary.pdf]

Journal of Fungi

Supplementary material

Impacts of biogas slurry fertilization on arbuscular mycorrhizal fungal communities in rhizosphere soil of poplar plantations

Running title: Effects of BS fertilization on AMF

Xing-Ye Yu[1], Bao-Teng Wang[1], Long Jin[1], Hong-Hua Ruan[1], Hyung-Gwan Lee[2,*], and Feng-Jie Jin[1,*]

[1] College of Biology and the Environment, Co-Innovation Center for Sustainable Forestry in Southern China, Nanjing Forestry University, 159 Longpan Road, Nanjing 210037, China

[2] Cell Factory Research Centre, Korea Research Institute of Bioscience & Biotechnology (KRIBB), Daejeon 34141, Republic of Korea

* Correspondence: jinfj@njfu.edu.cn (F.J.J.); trustin@kribb.re.kr (H.G.L.)

Tel.: +86-25-8542-7210 (F.J.J.); Tel.: +82-42-860-4318 (H.G.L.)

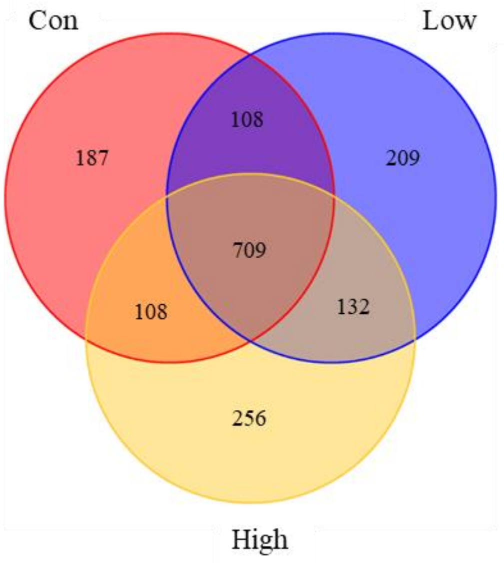

Fig. S1 Venn diagram depicting the relationships between treatments. The overlapped and unique OTU numbers of AMF communities in rhizospheric soil between treatments are shown in the Venn diagram.

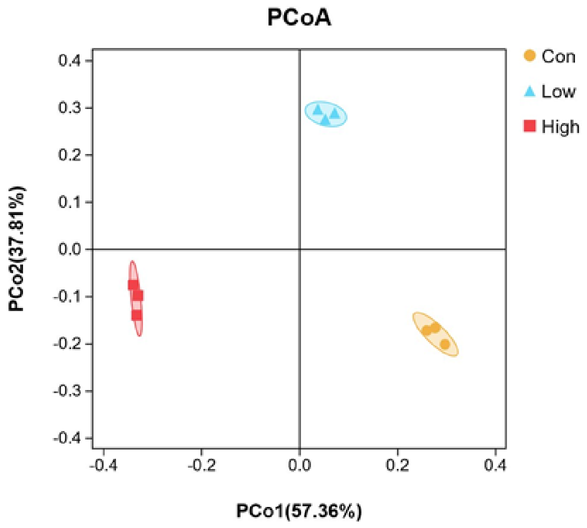

Fig. S2 Principal coordinates analysis on AMF community structure in rhizosphere soil of poplar plantations treated with different concentrations of biogas slurry. Con - no treatment; Low - biogas slurry (250 m^3 ha^{-1} yr^{-1}) treatment; High - biogas slurry (375 m^3 ha^{-1} yr^{-1}) treatment.

Table S1 Effects of biogas slurry treatments on the physical, chemical, and biological characteristics of rhizospheric soil (Yu et al., 2022[a])

Attribute	Con	Low	High
pH	7.90 ± 0.02 ab	7.80 ± 0.02 b	8.00 ± 0.02 a
Moisture content	0.20 ± 0.00 a	0.20 ± 0.00 a	0.21 ± 0.01 a
Total C (g kg^{-1})	18.60 ± 0.37 a	19.35 ± 0.31 a	19.28 ± 0.88 a
Total N (g kg^{-1})	1.22 ± 0.03 a	1.32 ± 0.08 a	1.35 ± 0.08 a
C / N	15.22 ± 0.15 a	14.74 ± 0.69 ab	14.28 ± 0.22 b
Nitrate (mg kg^{-1})	33.11 ± 3.69 b	50.58 ± 30.77 a	33.82 ± 4.24 b
Available P（mg kg^{-1})	2.65 ± 0.06 b	4.45 ± 0.49 a	5.07 ± 0.66 a
Microbial biomass C (mg kg^{-1})	240.45 ± 14.11 a	203.46 ± 25.78 a	153.16 ± 38.41 a
Microbial biomass N (mg kg^{-1})	15.52 ± 4.43 a	18.11 ± 1.90 a	19.12 ± 2.56 a
Phosphatase (mg kg^{-1})	1.49 ± 0.05 a	1.62 ± 0.06 a	1.50 ± 0.10 a
Nitrate reductase (μmol $d^{-1}g^{-1}$)	7.03 ± 1.12 ab	7.58 ± 0.09 b	11.36 ± 0.96 a

Values are Mean ± standard error (n=3); Different lowercase letters within the same row denote significant ($P < 0.05$) differences between treatments. Con - no treatment; Low - biogas slurry (250 m^3 ha^{-1} yr^{-1}) treatment; High - biogas slurry (375 m^3 ha^{-1} yr^{-1}) treatment.

[a] Yu, X.Y.; Zhu, Y.J.; Jin, L.; Wang, B.T.; Xu, X.; Zou, X.M.; Ruan, H.H.; Jin, F.J. Contrasting responses of fungal and bacterial communities to biogas slurry addition in rhizospheric soil of poplar plantations. Appl Soil Ecol 2022, 175, doi: 10.1016/j.apsoil.2022.104427.

Table S2 Effect of biogas slurry fertilization on α-diversity of AMF communities

Group	Shannon (P)	Chao1 (P)	No. of OTUs (P)	Phylogenetic diversity (P)
Con vs. Low	**0.0041**	0.1070	0.0884	0.1242
Con vs. High	**0.0442**	**0.0498**	0.0886	0.2746
Low vs. High	**0.0027**	0.2326	0.6793	0.2508
Con vs. Low vs. High	**0.0040**	**0.0250**	0.1680	0.2430

Significant P values ($P < 0.05$) are shown in bold. Con - no treatment; Low - biogas slurry (250 m^3 ha^{-1} yr^{-1}) treatment; High - biogas slurry (375 m^3 ha^{-1} yr^{-1}) treatment.

Table S3 Genetic differentiation of AMF communities between groups by analysis of molecular variance (AMOVA)

Group	*df*	*MS*	*F*	*P*
Con vs. Low	1	0.508473	24.2658	0.09
Low vs. High	1	0.434046	23.9339	0.094
Con vs. High	1	0.426067	23.1497	**0.019**
Con vs. Low vs. High	2	0.456195	23.8038	**0.001**

Significant *P* values ($P < 0.05$) are shown in bold. Con - no treatment; Low - biogas slurry (250 m^3 ha^{-1} yr^{-1}) treatment; High - biogas slurry (375 m^3 ha^{-1} yr^{-1}) treatment.

Table S4 Relative abundance of AMF community composition at the order level.

Order	Con	Low	High
Glomerales	0.400434 ± 0.012410 ab	0.362270 ± 0.013430 b	0.426858 ± 0.007948 a
Paraglomerales	0.062060 ± 0.001531 c	0.129547 ± 0.003331 a	0.106767 ± 0.001219 b
Diversisporales	0.040220 ± 0.001292 a	0.022593 ± 0.000878 b	0.038234 ± 0.001118 a
Archaeosporales	0.021925 ± 0.001935 a	0.005373 ± 0.000792 b	0.006036 ± 0.000717 b
Others	0.475361 ± 0.016947 a	0.480217 ± 0.016664 a	0.422104 ± 0.008927 a

Values are Mean ± standard error (n=3); Different lowercase letters within the same row denote significant ($P < 0.05$) differences between treatments. Con - no treatment; Low - biogas slurry (250 m^3 ha^{-1} yr^{-1}) treatment; High - biogas slurry (375 m^3 ha^{-1} yr^{-1}) treatment.

Table S5 Relative abundance of AMF community composition at the genus level.

Genus	Con	Low	High
Glomus	0.216391 ± 0.009579 b	0.241247 ± 0.007182 b	0.291485 ± 0.004839 a
Paraglomus	0.062060 ± 0.001531 c	0.1295476 ± 0.003331 a	0.106767 ± 0.001219 b
Claroideoglomus	0.100813 ± 0.003907 a	0.052352 ± 0.002110 b	0.086393 ± 0.002479 a
Diversispora	0.011904 ± 0.000561 a	0.009322 ± 0.000499 b	0.011917 ± 0.001088 ab
Scutellospora	0.006199 ± 0.000487 b	0.000149 ± 0.000011 c	0.016154 ± 0.000864 a
Archaeospora	0.007443 ± 0.000426 a	0.002596 ± 0.000643 b	0.000551 ± 0.000148 b
Ambispora	0.000591 ± 0.000421 b	0.000685 ± 0.000276 b	0.002418 ± 0.000280 a
Pacispora	0.000048 ± 0.000035 b	0.000003 ± 0.000003 b	0.000251 ± 0.000034 a
Redeckera	0.000206 ± 0.000047 a	0.000024 ± 0.000010 b	0.000022 ± 0.000009 b

Values are Mean ± standard error (n=3); Different lowercase letters within the same row denote significant ($P < 0.05$) differences between treatments. Con - no treatment; Low - biogas slurry (250 m^3 ha^{-1} yr^{-1}) treatment; High - biogas slurry (375 m^3 ha^{-1} yr^{-1}) treatment.
